# Differential associations of mental health, mild traumatic brain injury and substance use between male and female university students

**DOI:** 10.1371/journal.pone.0346562

**Published:** 2026-04-07

**Authors:** Alyssia Wilson, Jared Cherry, Kristina Gicas, Magdalena Wojtowicz

**Affiliations:** 1 Department of Psychology, York University, Toronto, Ontario, Canada; 2 Department of Neurology, Division of Movement Disorders, Yale University School of Medicine, New Haven, Connecticut, United States of America; 3 Department of Psychology, Rosalind Franklin University of Medicine and Science, North Chicago, Illinois, United States of America; 4 Department of Psychology, University of the Fraser Valley, Abbotsford, British Columbia, Canada; University of Toronto, CANADA

## Abstract

**Background:**

More severe substance use, defined as higher scores on validated measures of problematic use, is increasing within young adult populations. Substance use has been associated with mild traumatic brain injury (mTBI) and elevated symptoms of depression and anxiety. We aimed to understand the relationship between these factors and sex differences in a university sample.

**Method:**

894 university students (372 males and 522 females, aged 18–25 years) self-reported their mTBI history, substance use (Alcohol Use Disorders Identification Test (AUDIT) and the Cannabis Use Disorders Identification Test-Revised (CUDIT-R)), and psychological measures (Patient Health Questionnaire–9 (PHQ-9) and Generalized Anxiety Disorder–7 (GAD-7). Regression analyses examined whether the number of previous mTBIs was associated with increased cannabis (within the past six months) or alcohol use (within the past twelve months) severity. Logistic and linear regression models were used to explore how mTBI history, sex, and mental health symptoms relate to the likelihood and severity of cannabis and alcohol use and were also run separately by sex.

**Results:**

Individuals with multiple mTBIs reported more problematic substance use and a higher number of substances used. Hazardous substance use, as defined by scores above AUDIT and CUDIT-R cut-offs, was associated with both a previous history of mTBI and greater scores on depression and anxiety measures. Individuals with higher scores on an anxiety measure were more likely to use cannabis, especially if they had a history of mTBI. A history of mTBIs and higher current depression scores among cannabis users was associated with more problematic cannabis use. Among alcohol-using individuals, those with higher depression scores in addition to a history of mTBI were more likely to endorse more problematic alcohol use. Finally, males and females were affected differently by mental health and mTBI risk factors.

**Conclusion:**

mTBI history and mental health problems may be associated with hazardous substance use, but these findings highlight the importance of considering sex-specific risk patterns when developing interventions or preventive strategies for young adults with a history of mTBI or elevated anxiety/depression symptoms.

## Introduction

Substance use is rising and becoming more common among emerging adults, typically defined as those aged 18–29 [1], with half of substance-using Canadian youth engaging in polysubstance use. Use of substances, and in particular alcohol and cannabis, is complex and carries significant treatment and health implications [2–6]. Alcohol and cannabis use and hazardous drug use behaviours, as indicated by higher scores on substance use measures, among emerging adults are associated with negative health outcomes, including poor mental health [7–12]; however, the directionality of this relationship is highly debated [13]. Substance use and mental health disorders share environmental and genetic risk factors, increasing the likelihood of experiencing both [14,15]. For example, the self-medication hypothesis suggests that substance use is a coping mechanism for psychological stressors [14], whereas substance use and its sequelae may also induce additional mental health problems [15].

A history of mild traumatic brain injury(mTBI), as defined as a head injury resulting in loss of consciousness for less than 30 minutes, post-traumatic amnesia lasting less than 24 hours, and a Glasgow Coma Scale score of 13–15, has also been suggested as a predisposing risk factor for substance use and mood problems; however, more research is needed to better understand the mechanisms driving the relationships and their directionality [16–19]. Disruptions to neural pathways resulting from an mTBI may affect frontal brain regions involved in decision-making [20] and emotional regulation [21], which, in turn, may increase vulnerability to substance misuse as well as the increased likelihood of mental health problems. The relationship between mTBI, substance use, and mental health is complex, with prior research demonstrating that both mTBI and mental health uniquely contribute to substance use habits [22]. Further, while disruption of brain regions mediating risk-taking behaviour are proposed to contribute to increased substance use, it is also suggested that mTBI may influence substance use habits indirectly via mood disorders [23]; for example, individuals with mTBI who develop symptoms of depression or anxiety may engage in increased alcohol or cannabis use as a means of coping with emotional distress or physical and cognitive changes.

Untangling the associations between health factors and substance use in young adulthood is critical for early intervention and reducing the likelihood of continued substance use into later adulthood. In developing effective intervention programs, it is also important to consider how these factors may differentially affect males and females. Sex differences have been noted across substance use, mental health problems such as depression and anxiety, and mTBI. For example, adolescent males are more likely to engage in substance use [24] and transition to more risky substance use behaviour, including earlier onset, higher frequency of use, polysubstance use, and use of illicit drugs, than females [24,25], whereas females are more likely to suffer from mental health problems such as depression and anxiety [26]. Females may also have increased vulnerability to sustaining an mTBI [i.e., may be more likely to suffer an mTBI from a similar head impact due to physical differences, such as smaller neck size supporting head during impact, and hormonal differences] and research shows that females typically experience greater symptomology and may have longer recovery trajectory following an mTBI [27–33]. Recent literature has also shown that females with mTBI, especially those with repetitive injuries, reported significantly higher symptom burden, including post-concussive, PTSD, and anxiety symptoms, compared to men [34]. Despite sex differences in these risk factors, there is a lack of individualized treatment for males and females. Current treatment for mTBI typically involves rest, gradual return to activity, and symptom monitoring, but these approaches are generally standardized and do not account for sex-based differences in symptom presentation or recovery trajectories [35].

The aim of this study was to investigate how mental health and mTBI history are associated with recent patterns of substance use using regression models. Given the established sex differences in both substance use patterns and mental health outcomes, as well as possible differences in vulnerability to a recovery from mTBI, it is crucial to examine whether these relationships differ between males and females. To address this, we specifically used sex-based models to explore potential sex-specific variations in these interactions.

## Methods

### Participants

Ethics was approved by the Human Participants Review Sub-Committee of York University’s Ethics Review Board. Data collection began on September 1, 2025, and written informed consent was obtained through the digital questionnaire. Data were collected from 894 York University students through the undergraduate research participant pool in psychology or kinesiology who received partial course credit for their participation. Participants were eligible if: 1) they were between the ages of 17 and 25 and 2) were registered students in the Introduction to Psychology course.

### Measures

#### Alcohol use disorder identification test (AUDIT).

The AUDIT [36] is a 10-item screening index that was used to assess alcohol use in the past year. This questionnaire was designed for self-administration and is scored by adding each of the 10 items: questions 1–8 are scored on a 0–4 scale, questions 9 and 10 are scored 0, 2 or 4. Total scores of 0–7 indicate low risk, 8–15 indicate medium risk, 16–19 indicate high risk, and 20–40 points indicates likely alcohol addiction. Given that a large proportion of our sample indicated abstinence from alcohol in the past year, participants were considered alcohol users if they indicated any alcohol use in the past year. To better understand factors contributing to the degree of alcohol use, some analyses looked at total AUDIT scores solely within the alcohol use group.

#### Cannabis use disorder identification test-revised (CUDIT-R).

The CUDIT-R [37] is an 8-item screening index used to assess cannabis use in the past six months that can be administered to individuals who indicate cannabis use within the last six months. This questionnaire was designed for self-administration and is scored by adding each of the 8 items: questions 1–7 are scored on a 0–4 scale, question 8 is scored 0, 2, or 4. Scores of 8 or more indicate hazardous cannabis use, while scores of 12 or more indicate a possible cannabis use disorder for which further intervention may be required. Participants were considered cannabis users if they indicated any use of cannabis in the past six months. To better understand factors contributing to the degree of cannabis use, some analyses looked at total CUDIT-R scores solely within the cannabis use group.

#### Substance use history.

Participants answered questions about their substance use history, which inquired about current use of various substances in the past year (“Have you ever used ____” and “How often have you used ____ in the past year”) including tobacco/nicotine (including vaporizers), prescribed opioids/opioids in general, stimulants (e.g., cocaine, Adderall), steroids, and “other”. To provide data for an exploratory analysis of “polysubstance use”, the number of categories of substances used was included as a variable by summing the number of different types of substances used in the past year, including the above categories as well as cannabis and alcohol.

#### Patient health questionnaire-9 item (PHQ-9).

The PHQ-9 [38] is a screening tool derived from the Patient Health Questionnaire. The PHQ-9 is a brief self-report measure that evaluates 9 criteria for depressive disorders based on DSM-IV criteria. This self-administered tool assesses how often individuals were bothered by items over the last two weeks. Questions are scored on a 4-point ordinal scale (0; not at all, 1; several days, 2; more than half the days, 3; nearly every day). Total scores of 0–4 indicate none, 5–9 indicate mild depression, 10–14 indicate moderately severe depression, and 20–47 indicate severe depression. Possible major depressive disorder (MDD) was characterized by 5 or more items endorsed at least “more than half the days” or if items 1 or 2 are endorsed as at least “more than half the days”.

#### Generalized anxiety disorder-7 item (GAD-7).

The GAD-7 [39] is a well-validated, 7-item questionnaire to assess self-reported anxiety. This self-administered tool assesses how often individuals were bothered by items over the last two weeks. Questions are scored on a 4-point ordinal scale (0; not at all, 1; several days, 2; more than half the days, 3; nearly every day). Total scores of 0–4 indicate minimal anxiety, 5–9 indicate mild anxiety, 10–14 indicate moderate anxiety, and 15–21 indicate severe anxiety.

#### mTBI history.

Participants were asked questions about their mTBI history. An operational definition of a concussion was provided, as follows, “For this section, we define a concussion as a blow to the head or whiplash that caused any one or more of the following: witnessed loss of consciousness (LOC) (being “knocked out”, and someone saw it), loss of memory for events immediately before and/or after the injury (PTA), or feeling dazed and confused for at least 30 seconds. Participants indicated whether they had sustained zero, one, two, three, four, or five or more mTBIs. Participants provided dates (month and year) for each injury. Each identified injury was followed up by a series of questions asking: i) Did someone see you lose consciousness? ii) Were you dazed and confused? iii) Did you have no memory for events immediately after the injury? iv) Did you go to the hospital? v) Were you medically diagnosed with a concussion or brain injury? vi) Did you miss any school or work because of this injury? vii) Did you have symptoms for more than 24 hours? viii) Did you have symptoms for more than one week? ix) Did you have symptoms for more than one month?

#### Clinical history.

Information was also collected about years of education, endorsement of any previous diagnoses of anxiety, or depression, all through self-report. Rates of Attention-deficit/hyperactivity disorder (ADHD) and learning disorders were also collected but not included in main analyses.

### Procedures

Substance use measures, mental health measures, as well as information about mTBI and sport history, and demographic information were collected via an online survey (Redcap). Data were collected from September 2022 to November 2022 as part of the fall course requirement. Individuals who agreed to participate were required to complete the survey in a quiet, distraction-free environment.

### Statistical analyses

Data were analyzed using the Statistical Package for the Social Sciences (SPSS) version 28 (IBM Corp. Released, 2015). Although our questionnaire targeted mTBI history, some participants may have reported more severe injuries. We identified such cases (i.e., endorsement of both LOC and PTA, LOC and admission to the hospital, or LOC and an injury occurring due to a motor vehicle accident (n = 82)) and compared substance use outcomes with those reporting less severe mTBIs. As no group differences emerged, all participants were included in analyses. Independent t-tests were run where homogeneity of variance was met. T-tests and chi-square tests were used to analyze sex differences in AUDIT, CUDIT-R, GAD-7, and PHQ-9 scores and to compare differences between GAD-7 and PHQ-9 scores in females and males with and without a history of mTBI.

Binary regressions were used to compare mTBI history and cannabis use or alcohol use. Substance use and mental health was compared in those with one versus multiple mTBIs and linear regression was used to determine if sustaining one, two, or three or more mTBIs compared to no mTBIs, was significantly associated with increased scores on the CUDIT-R or AUDIT among cannabis and alcohol users group, or with the number of different types of substances used in the past year.

Binary regressions were used to compare mTBI history and cannabis use or alcohol use as well as hazardous and non-hazardous use and to compare mTBI in hazardous users with co-occurring moderate anxiety or depression scores.

A binary logistic regression model was used to look at whether endorsement of past mTBIs (none versus any), sex at birth, current psychological symptoms of depression or anxiety (PHQ-9, GAD-7 total scores) and the their interactions (i.e., mTBI history with sex, depression scores, or anxiety scores) were associated with the likelihood of use versus non-use of cannabis or alcohol (yes/no) among all participants. The same predictors were used in a linear regression to examine their relationship with total CUDIT-R and AUDIT scores among the cannabis and alcohol users group. The binary logistic regression models described above were run separately for males and females to identify substance use factors that may be obscured in combined models.

For each analysis, we investigated the extent to which the data met the assumptions of linearity, normality, and homoscedasticity of errors, as well as whether there was multicollinearity where relevant. Histograms and plots of studentized residuals were examined, and some mild violations were noted but did not warrant corrections or prevent the analyses. No participants were excluded for missing data.

## Results

### Sample characteristics

Demographic information is shown in Table 1. This sample was made up of 894 individuals. Sex at birth was 72.9% (N = 652) female, which was used in all analyses as our sample did not allow for a more in-depth sample of gender identity. Three hundred and twenty mTBIs were reported among 200 participants. Clinical information is shown in Table 2.

**Table 1:** Demographic information.

Variable	
Age in years M(SD)	19.0 (1.5)
Range	17-25
Gender n (%)	
Female	647 (72%)
Male	241(27%)
Non-binary	3 (0.3%)
Prefer to self-describe or not disclose	2 (0.2%)
Female sex at birth n (%)	652 (72.9%)
Male sex at birth n (%)	242 (27.1%)
Race	
Asian	364 (40.72%)
White	217 (24.27%)
Middle Eastern	104 (11.63%)
Black or African American	85 (9.51%)
South Asian	49 (5.48%)
Mixed Ethnicity	30 (3.36%)
Hispanic, Latino, or Latina	29 (3.24%)
American Indian or Alaska Native	3 (0.34%)
Native Hawaiian or Pacific Islander	2 (0.22%)
Prefer to not disclose	11 (1.23%)
Years of university education M (SD) Range	1.63 (.86) 1-6

Abbreviations: M = mean, SD = standard deviation.

**Table 2:** Clinical characteristics.

Cannabis use past six months n (%)		233 (26.05%)
CUDIT-R total M(SD)		2.07 (4.55)
Range		0-32
Hazardous cannabis use (over 8) n (%)		101 (11.30%)
Alcohol use past year n (%)		481 (53.80%)
AUDIT-C total M(SD)		1.99 (3.38)
Range		0-23
Hazardous alcohol use (over 8) n (%)		72 (8.05%)
PHQ9 total M(SD)		9.65 (6.33)
Range		0-27
PHQ9 mild or above n (%)		687 (76.85%)
PHQ9 moderate or above n (%)		407 (45.53%)
Possible MDD based on PHQ9 n (%)		369 (41.28%)
GAD7 total M(SD)		8.45 (5.87)
Range		0-21
GAD7 mild or above n (%)		619 (69.34%)
GAD moderate or above n (%)		351 (39.26%)
Hazardous cannabis use and PHQ9 moderate or above		59 (6.6%)
Hazardous cannabis use and GAD7 moderate or above		51 (5.7%)
Hazardous alcohol use and PHQ9 moderate or above		43 (4.8%)
Hazardous alcohol use and GAD7 moderate or above		41 (4.6%)
Previous diagnosis of anxiety n (%)		166 (18.57%)
Previous diagnosis of depression n (%)		116 (12.98%)
Tobacco use this year n (%)		231 (25.84%)
Opioid use this year n (%)		12 (1.34%)
Simulant use this year n (%)		27 (3.02%)
Steroid use this year n (%)		7 (0.78%)
Other drug use this year n (%)		39 (4.36%)
Number of substances used this year n (%)	0	338 (37.81%)
	1	257 (28.75%)
	2	160 (17.90%)
	3	112 (13.31%)
	4	19 (2.13%)
	5	7 (0.78%
	6	1 (0.11%)
Number of mTBIs endorsed across the lifetime n (%)	0	694 (77.62%)
	1	125 (13.98%)
	2	47 (5.26%)
	3	16 (1.79%)
	4	7 (0.78%)
	5 or more	5 (0.56%)
Time since last injury in years M(SD)		3.74(3.84)
Range		0-18
ADHD n (%)		48 (5.37%)
Learning disorder n (%)		39 (4.36%)

Abbreviations: AUDIT = Alcohol Use Disorder Identification Test, CUDIT-R = Cannabis Use Disorder Identification Test-Revised, GAD7 = Generalized anxiety disorder-7 item, M = mean, MDD = major depressive disorder, mTBI = mild traumatic brain injury, PHQ9 = Patient Health Questionnaire-9 item, SD = standard deviation.

### Associations between mTBI and mental health on substance use outcomes by sex

There were no significant differences in the proportions of males and females who used cannabis (*X*^2^([1], N = 894) =.487, *p* = [.485]), alcohol (*X*^2^([1], N = 894) =.877, *p* = [.349])), in overall CUDIT-R or AUDIT scores (t(892) = −.094, *p* = .925; t(892) =.893, *p* = .372), or whether they endorsed a history of mTBI (*X*^2^([1], N = 894) =.862, *p* = [.353]). However, females had significantly higher symptoms of depression and anxiety than males (t(892) = 7.428, *p* = .004; t(892) = 8.309, *p* = < .001; See Table 3). Additionally, current depression and anxiety symptom scores were significantly higher in females who endorsed a history of mTBI (M = 12.93, SD = 6.91; M = 11.50, SD = 6.07) compared to those who did not (M = 9.87, SD = 6.04; M = 8.78, SD = 5.70; t(650) = −5.276, *p* = .001; t(650) = −5.057, *p* = < .001). Whereas current depression and anxiety symptom scores did not differ for males with a history of mTBI (M = 8.37, SD = 5.77; M = 6.92, SD = 4.68), versus those without (M = 6.83, SD = 5.37; M = 5.60, SD = 5.04); t(240) = −1.763, *p* = .079; t(240) = −1.658, *p* = .099).

**Table 3:** Substance use, mTBI, and mental health demographics by sex.

	Female	Male	*p*-value
Cannabis use N(%)	174 (26.7%)	59 (24.4%)	.485
CUDIT-R total M(SD)	2.06 (4.49)	2.10 (4.71)	.925
Alcohol use N(%)	357 (54.8%)	124 (51.2%)	.349
AUDIT total M(SD)	2.36 (3.37)	2.14 (3.16)	.372
mTBI history N(%)	151 (23.2%)	49 (20.2%)	.353
PHQ9 total M(SD)	10.57 (6.38)	7.14 (5.48)	.004**
GAD7 total M(SD)	9.41 (5.89)	5.87 (4.99)	<.001***

Note: *p < .05. **p < .01. ***p < .001

Abbreviations: AUDIT = Alcohol Use Disorder Identification Test, CUDIT-R = Cannabis Use Disorder Identification Test-Revised, GAD7 = Generalized anxiety disorder-7 item, M = mean, mTBI = mild traumatic brain injury, PHQ9 = Patient Health Questionnaire-9 item, SD = standard deviation.

### Associations between mTBI on substance use outcomes

Binary logistic regressions indicated that mTBI history was not a significant predictor of cannabis use, (B=0.224, SE = 0.178, Wald(1) = 1.576, p = .209, odds ratio = 1.25) and that mTBI history significantly as associated with a greater likelihood of alcohol use (B=0.325, SE = 0.163, Wald(1) = 3.963, p = .047, odds ratio = 1.38). Among those with previous mTBI, participants with a history of two or more mTBIs (n = 75) versus only one mTBI (n = 125) reported significantly higher scores on cannabis use (CUDIT: M = 4.04 vs. 2.31; t(198) = –2.05, *p* = .042), alcohol use (AUDIT: M = 3.75 vs. 2.39; t(198) = –2.37, *p* = .019), anxiety symptoms (GAD-7: M = 12.55 vs. 9.07; t(198) = –4.06, *p* < .001), and depressive symptoms (PHQ-9: M = 14.39 vs. 10.27; t(198) = –4.24, *p* < .001).

To investigate further whether the number of past mTBIs (i.e., one, two, or three or more), is associated with the severity of substance use within users, separate regressions were run for cannabis users (N = 233) and alcohol users (N = 481). Among cannabis users, having two (b = .194, t(229) = 3.01, *p* = .003) or three or more mTBIs (b = .162, t(229) = 2.52, *p* = .013), but not one (b = .093, t(229) = 1.43, *p* = .154), was associated with increased cannabis use. For alcohol users, only those with three or more mTBIs showed higher alcohol use (b = .208, t(477) = 4.61, p < .001). Exploratory analyses, showed that sustaining two sustaining two (b = .067, t(890) = 2.02, *p* = .044) or three or more mTBIs (b = .180, t(890) = 5.44, *p* < .001), was associated with a greater *number* of different substances used.

Those with hazardous cannabis use were also more likely to report moderate or greater anxiety and depression (GAD-7: 50.5% vs. 37.8%, χ²(1, *N* = 894) = 6.03, *p* = .014; PHQ-9: 58.4% vs. 43.9%, χ²(1, *N* = 894) = 7.63, *p* = .006). Similarly, individuals with hazardous alcohol use were more likely to report moderate or greater anxiety and depression (GAD-7: 56.9% vs. 37.7%, χ²(1, *N* = 894) = 10.27, *p* = .001; PHQ-9: 59.7% vs. 44.3%, χ²(1, *N* = 894) = 6.36, *p* = .012). Binary logistic regressions show that mTBI history was significantly associated with hazardous cannabis use (B=0.492, SE = 0.233, Wald(1) = 4.473, p = .034, odds ratio = 1.64), and hazardous alcohol use (B=0.537, SE = 0.267, Wald(1) = 4.053, p = .044, odds ratio = 1.71).

A post-hoc analysis showed that individuals with both hazardous alcohol use and moderate or greater anxiety (GAD-7; *n* = 41) were more likely to report a history of mTBI (41.5%) than those without co-occurring challenges (21.5%; χ²(1, *N* = 894) = 9.02, *p* = .003). A similar pattern was found for those with hazardous alcohol use and moderate or greater depression (PHQ-9; *n* = 43; 41.9% vs. 21.4%; χ²(1, *N* = 894) = 9.88, *p* = .002). In contrast, hazardous cannabis use combined with moderate or greater anxiety (GAD-7; *n* = 51) was not linked to higher mTBI rates. However, those with hazardous cannabis use and moderate or greater depression (PHQ-9; *n* = 59) were more likely to report mTBI (33.9% vs. 21.6%; χ²(1, *N* = 894) = 4.83, *p* = .028).

### Associations between mTBI, mental health and sex on substance use outcomes

**Fig 1:**
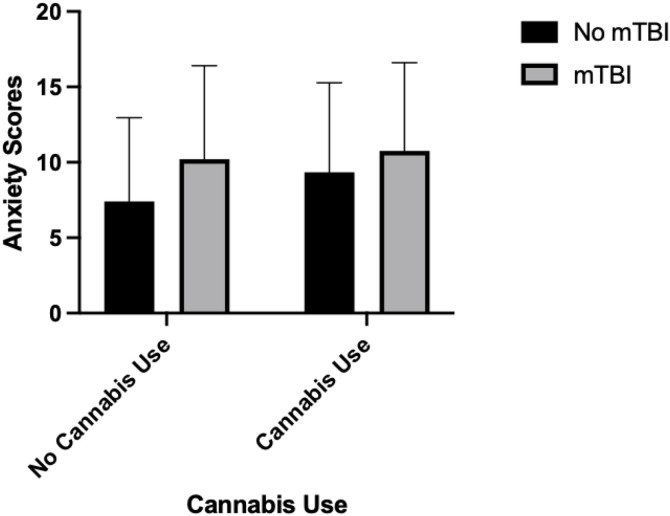
Interaction effects from a binary logistic regression showing mean GAD-7 anxiety scores (± SD) by cannabis use status and mTBI history (*p* =  .016). Note. Descriptive statistics are as follows: no cannabis/no mTBI (M = 7.41, SD = 5.54), no cannabis/mTBI (M = 10.22, SD = 6.19), cannabis/no mTBI (M = 9.34, SD = 5.94), and cannabis/mTBI (M = 10.75, SD = 5.86).

Binary logistic regressions were conducted to examine whether mTBI history (none versus any), sex, GAD-7 scores, PHQ-9 scores, and their interactions with mTBI were associated with cannabis or alcohol use versus non-use (see Table 4). For cannabis use, (χ2(7) = 22.72, *p* = .002), higher anxiety scores were associated with greater likelihood of cannabis use (OR= 1.056, 95% CI [1.008, 1.107], *p* = .022). A significant interaction term between mTBI history and anxiety indicated that higher anxiety was more strongly associated with cannabis use among individuals without an mTBI compared to those with an mTBI (OR=.016, 95% CI [.799,.977], *p* = .016; see Fig 1). None of these variables showed a relationship with alcohol use (χ2(7) = 12.578, *p* = .083). Table 4 summarizes estimates of the unstandardized parameters, standard errors, along with the associated Wald statistics, *p*-values, odds ratios and 95% confidence intervals.

**Table 4:** Multivariable binary logistic regressions showing the association between mTBI, mental health, and cannabis and alcohol use versus non-use for all participants (N = 894).

Predictors	B(SE)	Wald	*p*-value	*OR*	*95%CI OR*	*R* ^*2*^
Model 1: Cannabis use versus non-use						.025
mTBI history	.326(.437)	0.556	.456	1.385	0.588, 3.260	
Sex	−.064(.207)	0.096	.757	0.938	0.625, 1.408	
Depression symptoms	.006(.022)	0.068	.794	1.006	0.963, 1.051	
Anxiety symptoms	.055(.024)	5.266	.022*	1.056	1.008, 1.107	
mTBI history x sex	.013(.441)	0.001	.977	1.013	0.427, 2.406	
mTBI history x depression symptoms	.086(.045)	3.663	.056	1.090	0.998, 1.191	
mTBI history x anxiety symptoms	−.124(.051)	5.820	.016*	0.883	0.799, 0.977	
Constant	−1.560(.206)	57.480	<.001	0.210		
Observations	**894**					
Model 2: Alcohol use versus non-use						.014
mTBI history	.595(.387)	2.365	.124	1.814	0.849, 3.873	
Sex	.036(.176)	0.041	.840	1.036	0.734, 1.463	
Depression symptoms	.023(.020)	1.275	.259	1.023	0.983, 1.064	
Anxiety symptoms	.016(.021)	0.587	.444	1.016	0.975, 1.060	
mTBI history x sex	−.011(.396)	0.001	.978	0.989	0.455, 2.150	
mTBI history x depression symptoms	−.007(.040)	0.031	.861	0.993	0.919, 1.073	
mTBI history x anxiety symptoms	−.028(.045)	0.385	.535	0.973	0.891, 1.061	
Constant	−.278(.171)	2.637	.104	0.758		
Observations	**894**					

*p < .05. **p < .01. ***p < .001.

Abbreviations: CI = confidence interval, mTBI = mild traumatic brain injury, OR = odds ratio.

The same variables were used to look at the association with CUDIT-R total scores in cannabis users and AUDIT scores among alcohol users. See Table 5 for estimates of the unstandardized parameters, along with the associated *t* statistics, *p*-values and 95% confidence intervals. For cannabis users, the model (*R* ^2^ of.098 (*F*(7,225) = 3.486, *p* = .001)) showed significant associations with mTBI (b = .438, t(225) = 2.69, *p* = .008) and depression scores (b = 301, t(225) = 2.80, *p* = .006) indicating that individuals with prior mTBI or higher depression scores endorsed more severe cannabis use. For alcohol users, the model (*R* ^2^ of.050 (*F*(7,473) = 3.584, *p* < .001)) revealed a significant interaction between mTBI history and depression scores (b = .328, t(473) = 2.05, *p* = .041), such that increased symptoms of depression were associated with more problematic alcohol use among those with an mTBI history.

**Table 5:** Association between mTBI, mental health, and sex variables and total cannabis and alcohol use in users.

Predictors	Unstandardized estimate	95% CI	*t-*statistic	*p*-value
Model 1: Total CUDIT-R scores in users				
(Intercept)	5.702	3.647,7.757	5.468	<.001
mTBI history	5.754	1.534, 9.975	2.687	.008**
Sex	−1.398	−3.380, 0.583	−1.391	.166
Depression symptoms	0.286	0.085, 0.486	2.803	.006**
Anxiety symptoms	−0.038	−0.233, 0.157	−0.383	.702
mTBI history x sex	0.298	−3.679, 4.274	0.148	.883
mTBI history x depression symptoms	−0.349	−0.816, 0.118	−1.474	.142
mTBI history x anxiety symptoms	0.062	−0.460, 0.585	0.235	.815
Observations	**233**			
Model 2: Total AUDIT scores in users				
(Intercept)	3.332	2.491, 4.173	7.784	<.001
mTBI history	0.180	−1.584, 1.944	0.201	.841
Sex	0.177	−0.666, 1.019	0.412	.680
Depression symptoms	0.040	−0.054, 0.134	0.833	.405
Anxiety symptoms	0.025	−0.071, 0.122	0.516	.606
mTBI history x sex	−1.712	−3.444, 0.020	−1.943	.053
mTBI history x depression symptoms	0.187	0.008, 0.367	2.052	.041*
mTBI history x anxiety symptoms	−0.059	−0.259, 0.140	−0.585	.559
Observations	**481**			

Note: *p < .05. **p < .01. ***p < .001.

Abbreviations: CI= confidence interval, mTBI = mild traumatic brain injury.

**Fig 2:**
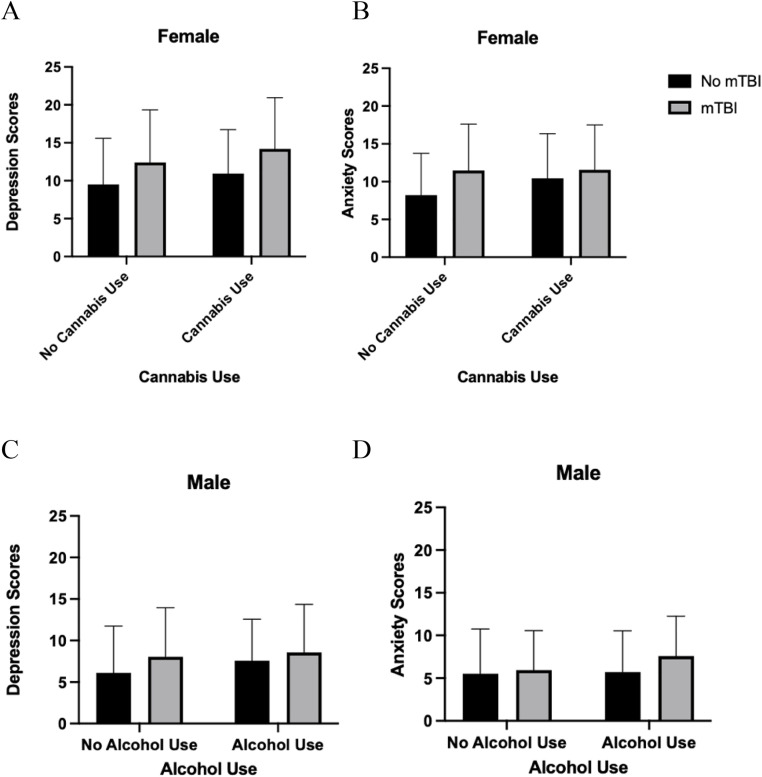
Interaction effects from a binary logistic regression model showing mean (± SD) depression (p =  .022) and anxiety (p =  .003) symptoms by cannabis use and mTBI in females and mean (± SD) depression (p =  .046) and anxiety (p =  .041) symptoms by alcohol use and mTBI in males. Note. Descriptive statistics are as follows A) mean PHQ-9 scores for females were: no cannabis/no mTBI (*M* = 9.50, *SD* = 6.09), no cannabis/mTBI (*M* = 12.38, *SD* = 6.97), cannabis/no mTBI (*M* = 10.82, *SD* = 5.80), and cannabis/mTBI (*M* = 13.91, *SD* = 6.53). B) Corresponding mean GAD-7 scores were: no cannabis/no mTBI (*M* = 8.21, *SD* = 5.52), no cannabis/mTBI (*M* = 11.50, *SD* = 6.17), cannabis/no mTBI (*M* = 10.40, *SD* = 5.86), and cannabis/mTBI (*M* = 11.34, *SD* = 5.85). C) Mean PHQ-9 scores for males were: no alcohol/no mTBI (M = 6.11, SD = 5.63), no alcohol/mTBI (*M* = 8.05, *SD* = 5.90), alcohol/no mTBI (*M* = 7.57, *SD* = 5.01), and alcohol/mTBI (*M* = 8.59, *SD* = 5.77). D) Corresponding mean GAD-7 scores were: no alcohol/no mTBI (M = 5.50, SD = 5.25), no alcohol/mTBI (*M* = 5.95, *SD* = 4.62), alcohol/no mTBI (*M* = 5.71, *SD* = 4.84), and alcohol/mTBI (*M* = 7.59, *SD* = 4.68).

Four binary logistic regressions tested whether mTBI history (none versus any), depression, anxiety scores, and their interactions were associated with substance use or non-use separately for females and males. For females (χ2(5) = 21.14, p < .001), those with higher anxiety scores were more likely to endorse cannabis use (OR= 1.088, 95% CI [1.032, 1.148], p = .022). Interaction effects showed that, higher depression scores were associated with greater likelihood of cannabis use among those with a history of mTBI (OR=1.126, 95% CI [1.017, 1.246], p = .022; see Fig 2A), whereas higher anxiety scores were associated with greater likelihood of cannabis use for those without a mTBI (OR=.843, 95% CI [0.753, 0.945], p = .003; see Fig 2B). For males, (χ2(5) = 8.28, p = .141), higher depression scores were associated with greater likelihood of cannabis use (OR= 1.112, 95% CI [1.007, 1.228], p = .036).

For alcohol use in females (χ2(5) = 10.71, *p* = .057) none of the predictors were associated with likelihood of use. For males (χ2(5) = 10.82, *p* = .055), higher depression scores were associated with greater likelihood of alcohol use (OR= 1.121, 95% CI [1.026, 1.224], *p* = .012). Interaction effects showed that, males with higher depression scores were less likely to use alcohol if they had a history of mTBI (OR=.832, 95% CI [0.694, 0.997], *p* = .046; see Fig 2C), whereas higher anxiety scores were associated with greater likelihood of alcohol use among males with a history of mTBI (OR= 1.258, 95% CI [1.010, 1.568], *p* = .041; See Fig 2D). Table 6 summarizes estimates of the unstandardized parameters (B), standard errors (SE), along with the associated Wald statistics, *p*-values, odds ratios (OR) and 95% confidence intervals (CI) for all of the above models.

**Table 6:** Association between mTBI, mental health, and cannabis and alcohol use verses non-use by sex.

Predictors	B(SE)	Wald	*p*-value	*OR*	*95%CI OR*	*R* ^*2*^
Model 1: Cannabis use versus non-use in females						.032
mTBI history	.464(.461)	1.016	.313	1.591	0.645, 3.925	
Depression symptoms	−.021(0.26)	0.681	.409	0.979	0.931, 1.030	
Anxiety symptoms	.085(.027)	9.600	.002**	1.088	1.032, 1.148	
mTBI history x depression symptoms	.118(.052)	5.258	.022*	1.126	1.017, 1.246	
mTBI history x anxiety symptoms	−.170(.058)	8.575	.003**	0.843	0.753, 0.945	
Constant	−1.628(.216)	56.944	<.001	0.196		
Observations	**652**					
Model 2: Cannabis use versus non-use in males						.034
mTBI history	−.020(.710)	0.001	.978	0.981	0.244, 3.946	
Depression symptoms	.106(.051)	4.378	.036*	1.112	1.007, 1.228	
Anxiety symptoms	−.057(.056)	1.046	.306	0.944	0.846, 1.054	
mTBI history x depression symptoms	−.033(.098)	0.113	.737	0.968	0.799, 1.172	
mTBI history x anxiety symptoms	.069(.119)	0.342	.559	1.072	0.850, 1.352	
Constant	−1.635(.295)	30.614	<.001	0.195		
Observations	**242**					
Model 3: Alcohol use versus non-use in females						.016
mTBI history	.692(.415)	2.776	.096	1.997	0.885, 4.507	
Depression symptoms	−.003(.023)	0.017	.897	0.997	0.953, 1.043	
Anxiety symptoms	.045(.025)	3.390	.066	1.046	0.997, 1.098	
mTBI history x depression symptoms	.038(.045)	0.731	.393	1.039	0.952, 1.134	
mTBI history x anxiety symptoms	−.088(.050)	3.074	.080	0.916	0.831, 1.010	
Constant	−.241(.178)	1.839	.175	0.786		
Observations	**652**					
Model 4: Alcohol use versus non-use in males						.044
mTBI history	.314(.597)	0.276	.599	1.369	0.425, 4.407	
Depression symptoms	.114(.045)	6.363	.012*	1.121	1.026, 1.224	
Anxiety symptoms	−.086(.048)	3.177	.075	0.918	0.836, 1.009	
mTBI history x depression symptoms	−.184(.092)	3.990	.046*	0.832	0.694, 0.997	
mTBI history x anxiety symptoms	.230(.112)	4.191	.041*	1.258	1.010, 1.568	
Constant	−.330(.240)	1.903	.168	0.719		
Observations	**242**					

*p < .05. **p < .01. ***p < .001.

Abbreviations: CI = confidence interval, mTBI = mild traumatic brain injury, OR = odds ratio.

## Discussion

This study aimed to examine the relationship between mTBI history with substance use (cannabis use within the past six months and alcohol use within the past year), as well as to understand the contribution of mental health to this relationship and sex related differences in these associations. Similar to prior research, we found an association between mTBI history and substance use in the past 6-months to one year [18]. Risk-taking behaviour such as problematic substance use is common following mTBI [40–42] and may be related to underlying neuropathology following head injury [42]. Our study suggests that the number of past injuries was an important factor to consider when examining problematic use of these substances It may be that while sustaining one injury is associated with current substance use in general, more severe use is associated with individuals who sustained multiple injuries. Specifically, sustaining one injury was not significantly associated with more severe current substance use in our study, however, sustaining two or more prior mTBIs was associated with more severe cannabis use and sustaining three or more previous injuries was associated with more severe alcohol use among users. Similarly to other studies [17,43], we found that sustaining multiple injuries was associated with a *greater number of substances* being used over the course of the past year. We also found that individuals with hazardous cannabis or alcohol use were more likely to have a history of mTBI and endorse current (i.e., within the last two weeks) moderate to severe depression and anxiety scores.

The relationship between increased risk of mental health problems following mTBI has been shown consistently in the literature [44–46], with a recent meta-analysis indicating that individuals with a history of mTBI, including both civilians and service members, are more than three times as likely to experience depression [47]. Furthermore, sustaining multiple mTBIs may place individuals at an even greater risk of experiencing depression [48]. The relationship between mTBI and depression is complex and likely affects substance use behaviours, while substance use also likely impacts mental health [13,17]. Recent research suggests that mTBI exposure has both direct and indirect effects on substance use via depression [23]. Similarly, our study showed that both endorsement of a previous mTBI and higher current depression scores were independently associated with more problematic cannabis use in cannabis users. Additionally, when examining problematic use in alcohol users, there was a small but significant interaction where increased depression scores in individuals with a past mTBI were associated with more problematic alcohol use. It may be that mTBI may interact by disrupting neural circuits involved in mood regulation, increasing vulnerability to depression and anxiety. These emotional difficulties may, in turn, contribute to substance use as individuals attempt to self-medicate or cope with negative affect.

The relationship between mTBI and anxiety has been less studied, but current findings show that college athlete alumni who have a history of mTBI endorse greater long-term anxiety symptoms [49] and one study examining adults following mTBI suggested that in the first few months following an mTBI, anxiety may be more prevalent than depression symptoms [50]. In fact, another recent study following adults who experienced mTBI shows that over 30% of individuals met the criteria for one or more anxiety disorders within a year of sustaining an mTBI and those with pre-injury anxiety were even more susceptible [51]. While anxiety and the use of alcohol and cannabis have been suggested to be associated via the self-medication model and the substance-induced model [52], additional endorsement of a previous mTBI may affect these models and may do so differently for these disorders. Consistent with previous literature, higher current anxiety symptoms were associated with an increased likelihood of cannabis use in our sample. Furthermore, anxiety symptoms interacted with mTBI history: among females, those with a history of mTBI exhibited significantly higher anxiety scores than those without a history of mTBI. Males showed the same pattern, despite not reaching significance. However, in individuals without a history of mTBI, higher anxiety scores were associated with greater likelihood of cannabis use. This aligns with other research showing associations between cannabis use and anxiety [53]. However, it also suggests a more nuanced relationship: anxiety is associated with cannabis use in individuals without an mTBI history, whereas in those with a history of mTBI, both anxiety and the injury itself may be linked to cannabis use. It should be noted that prior mental health diagnoses were not included in these models, which limits conclusions about the influence of pre-existing conditions on post-TBI outcomes.

The sex gap in the proportion of individuals with substance use disorders is narrowing [54] and women may be particularly at risk of experiencing higher rates of psychiatric disorders co-occurring with substance use disorders, which further complicates treatment [55]. Several studies have shown that women who use cannabis tend to report higher anxiety symptoms during treatment and withdrawal from use [56,57]. While research shows that depression strongly co-occurs with cannabis use, results investigating sex differences in depression among cannabis users are more mixed [58]. In our sample, females had significantly greater current mental health symptoms compared to males, and interactions between mental health and mTBI on cannabis use were observed exclusively in females. More specifically, in females, cannabis use was associated with both a prior mTBI history and higher current depression symptoms, while higher current anxiety symptoms appeared to be associated with cannabis use in those without an injury history. In contrast, higher current depression symptoms in males were associated with cannabis use. No associations between anxiety and cannabis use were observed in males in our sample. Our findings suggest that there may be differential relationships between mental health symptoms and cannabis use between sexes.

Furthermore, in our male sample, we observed that current mental health scores and mTBI history were associated with alcohol use. Alcohol use and risky use have become more normative particularly among university students [59], with about half our sample partaking in alcohol within the past year. The co-occurrence of anxiety and alcohol use [60] and depression and alcohol use [10,61], and increased susceptibility to depression following mTBI have been well established [62,63]. Likewise, in our sample, both depression and anxiety scores were higher—though not significantly—in males with mTBI compared to males without mTBI. However, higher anxiety scores, in addition to a history of mTBI were associated with greater alcohol use in males. Whereas none of these predictors were associated with alcohol use in females in our sample. This suggests that other factors including peer influencing, social norms, and coping styles among others, may contribute to alcohol use in females, whereas alcohol use in males might be related to their mental health and history of mTBI.

There are several limitations to this study. First, the participant pool was limited to psychology and kinesiology students at a single institution, which may affect the generalizability of these findings. Additionally, we cannot assume temporal causality with any of these relationships. Despite examining “past” mTBI and “current” substance use, we cannot control for the possibility of lifetime substance use that preceded any injuries. While self-reported prior mental health diagnoses were collected, we lacked details on the timing, duration, and severity of these conditions, limiting our ability to interpret their potential impact on substance use outcomes. As such, these findings should be interpreted as reflecting associations between these factors rather than causal relationships. The identification of mTBI history was based on a self-report questionnaire, and although a definition of mTBI was provided to participants, it is possible that this measure also captured more significant injuries. We attempted to address this by examining the characteristics of the reported injuries (see Analysis section) and found no differences in outcome measures for those who may have exhibited more significant signs or symptoms (e.g., LOC and PTA). Similarly, our study relied on self-reported substance use habits, which may be subject to reporting bias. Additionally, the smaller sample size of male participants led to wider confidence intervals and reduced statistical power, which may have limited our ability to detect significant effects in males and could have biased results toward patterns observed in the larger female sample.

## Conclusion

In conclusion, this study aimed to better understand the associations between mTBI and mental health on alcohol and cannabis use among young adults and to explore how these factors may be associated differently in males and females. We found that while mTBI and mental health were not significantly associated with alcohol use patterns over the past year in females, mTBI history in combination with either depression or anxiety symptoms was associated with cannabis use in females. In contrast, depression symptoms and mTBI history were correlated with alcohol and cannabis use in males. Our findings suggest that males and females may use substances for different reasons. Females may be more likely to use cannabis as a coping mechanism for anxiety, whereas depression may predispose males to alcohol or cannabis use in general. Additionally, individuals with mTBI tend to report more mental health problems. By recognizing these different factors in males and females, our findings support the need for more individualized treatment approaches for young adults.
